# The Last New Thing in Hospitals

**Published:** 1892-11-19

**Authors:** 


### PASSING TOPICS.

### THE LAST NEW THING IN HOSPITALS.

THE advertisement which is now going the round of the newspapers by way of an appeal on behalf of the "Hospital for Curing Loss of Movement from Paralysis, Rheumatism, &c.," is funny enough to raise a smile even in these *blasé* days. We have heard a great deal at one time and another about the multiplication of special hospitals and all its attendant evils, but the strictest stickler for legitimate business upon the hospital stage cannot refuse the tribute of his interest to the mirth-provoking burlesque which appears as the latest novelty upon the institutional playbill.

For our own part we have no hesitation about placing upon record our unstinted admiration for the talented author of this remarkable production. So far from being derived from the French or any Continental source, we venture to assert that the evidence of its English origin is overwhelming. In no other climate in the world but our own could the idea have blossomed and borne fruit, or even have appealed barrenly to public sympathy. There is a boldness, too, and an unflinching courage about the venture which we like to associate exclusively with English enterprise. Whether loss of movement comes from paralysis, rheumatism, or "et cetera"—be you the occupant of a private room at "from two guineas a week" or a mere out-patient —the result is to be identically the same. Three dances from nine to one in the Westminster Town Hall, the third in fancy dress—blissful consummation!—will reward your endeavours. As to this we have only to remark that we ourselves should be very well satisfied with a less liberal illustration of the results of treatment. To dance from nine to one is no doubt a sufficiently tempting, if somewhat distressing, prospect to a paralytic or rheumatic subject, and we would suggest that half an hour's exertion of the sort would be more than enough to convince the most sceptical that the power of movement had returned, and that the hospital which promised a cure had carried out its undertaking to the letter. It is, we suppose, permissible to hope that the therapeutical methods of this institution are not those of the celebrated doctor, who is said, if our memory is not treacherous, to have been:—

"A very good man,
Who whipped his patients now and then,
Whipped them till he made them dance
Out of England into France,
Out of France into Spain,
And then he whipped them back again."

Anything short of drastic treatment of that description would, no doubt, be patiently endured as the prelude to so radical a cure. Information upon the point would be welcome to many who have read the advertisement with a lively anticipation, and upon the assumption that it will prove satisfactory and reassuring, we recommend the Hospitals for Paralysis and the Bath Mineral Water Hospital to look to it that their reputation is not filched from them, and their claim to exist seriously imperilled. AGRICOLA.

